# Transcranial Color‐Coded Duplex Sonography in Large‐Vessel Vasculitis Compatible With Takayasu Arteritis Presenting Intracranial Involvement and Vertebrobasilar Dolichoectasia: A Case Report

**DOI:** 10.1002/jcu.70237

**Published:** 2026-04-01

**Authors:** Maria Júnia Lira e Silva, Jordana Gaudie Gurian, Isabella Mesquita Venâncio, Eva Carolina Rocha, Gisele Sampaio Silva

**Affiliations:** ^1^ Universidade Federal de São Paulo, (UNIFESP) São Paulo SP Brazil

**Keywords:** large‐vessel vasculitis, stroke, Takayasu arteritis, transcranial color‐coded duplex

## Abstract

Takayasu arteritis (TA) is a large‐vessel vasculitis that affects predominantly extracranial vessels. However, with advances in neuroimaging techniques, a better understanding of intracranial involvement has emerged. We describe the case of a 50‐year‐old woman with progressive large‐vessel disease and sequential intracranial occlusions, in whom TA was considered the most likely diagnosis after multidisciplinary evaluation. The transcranial color‐coded duplex (TCCD) sonography identified focal stenosis in the intracranial portion of the right internal carotid artery and collateralization through the circle of Willis. The collateral flow probably protected the patient from other ischemic events.

## Graphical abstract

This case illustrates sequential intracranial occlusions in large‐vessel vasculitis compatible with Takayasu arteritis preserving perfusion through robust collateral pathways. Transcranial color‐coded duplex sonography identified characteristic blunted flow patterns and focal turbulence, underscoring its value in detecting hemodynamic compromise beyond extracranial stenosis.

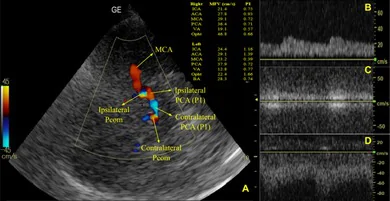

## 1 Introduction

Takayasu arteritis (TA) is a large‐vessel vasculitis characterized by granulomatous inflammation that primarily affects young women (Fauci 1994). While extracranial vessel involvement is well described, studies of the intracranial vasculature have often focused on the collateralization patterns that develop in response to proximal occlusion (Hoffmann et al. 2000). Advances in neuroimaging techniques have allowed better characterization of intracranial stenoses that may be responsible for ischemic events in TA (Bond et al. 2017). As a noninvasive and highly versatile imaging method, transcranial color‐coded duplex (TCCD) sonography is a tool capable of identifying both collateralization patterns and areas of probable focal stenosis.

Although computed tomography (CT) and magnetic resonance imaging (MRI) angiography remain the reference modalities for large‐vessel vasculitis, ultrasound plays a complementary role by providing real‐time assessment of vessel wall morphology and, importantly, functional and hemodynamic consequences of arterial involvement. Doppler techniques are particularly valuable for detecting altered flow patterns, collateral activation, and downstream perfusion changes, which may precede or not be fully captured by purely anatomical imaging. This functional perspective is especially relevant in large‐vessel diseases affecting the aorta and its major branches, such as TA (Germanò et al. 2017; Monti et al. 2018). We present the case and TCCD findings of a patient with sequential intracranial occlusions, most of which were asymptomatic, with collateral circulation maintained through the circle of Willis.

## 2 Case Report

**FIGURE 1:**
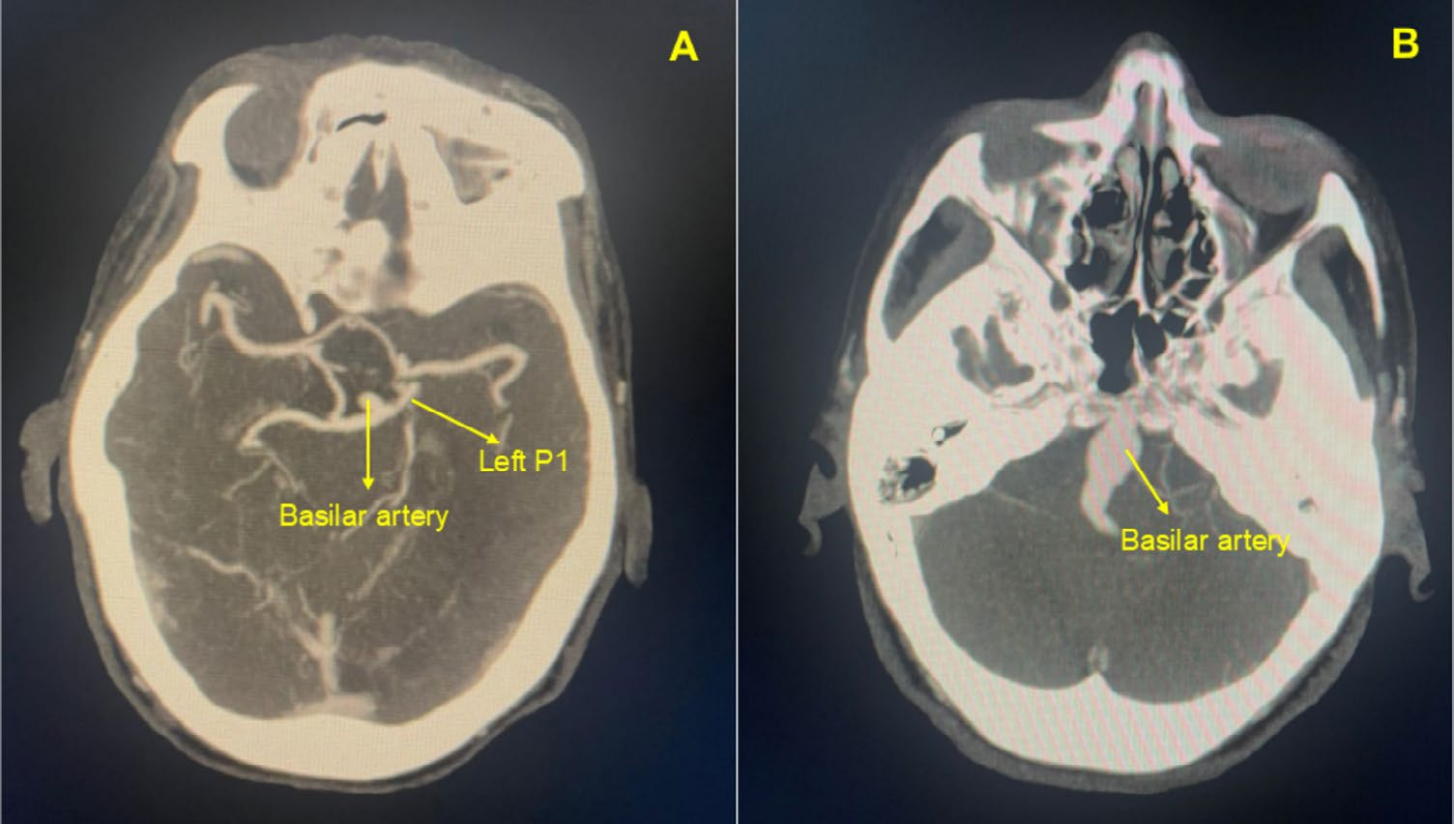
Axial computed tomography angiography image demonstrating an ectatic basilar artery with asymmetric configuration of the P1 segment of the PCA. The left P1 arises at an oblique angle, reflecting asymmetry in posterior circulation vascular anatomy (A). Severe basilar artery ectasia (B). PCA, posterior cerebral artery.

A woman at the age of 50 years, hypertensive and smoker, presented with her first stroke with P1 segment occlusion of the left posterior cerebral artery (PCA). At that time, given the patient's age and vascular risk factors, atherosclerosis was initially considered a relevant differential diagnosis. This was the only symptomatic event at presentation. She denied headaches, constitutional symptoms, or jaw pain. One year later, during reevaluation, computed tomography angiography (CTA) revealed occlusion of the right internal carotid artery (ICA) and subocclusion of the left ICA, in addition to tortuous vertebral and basilar ectasia (Figure 1).

The laboratory was unremarkable, with a normal erythrocyte sedimentation rate (ESR) (6 mm/h). Lipid profile obtained prior to lipid‐lowering therapy showed total cholesterol of 167 mg/dL, LDL cholesterol of 90 mg/dL, HDL cholesterol of 48 mg/dL, and triglycerides of 110 mg/dL. Lipoprotein(a) dosages were not available.

**FIGURE 2:**
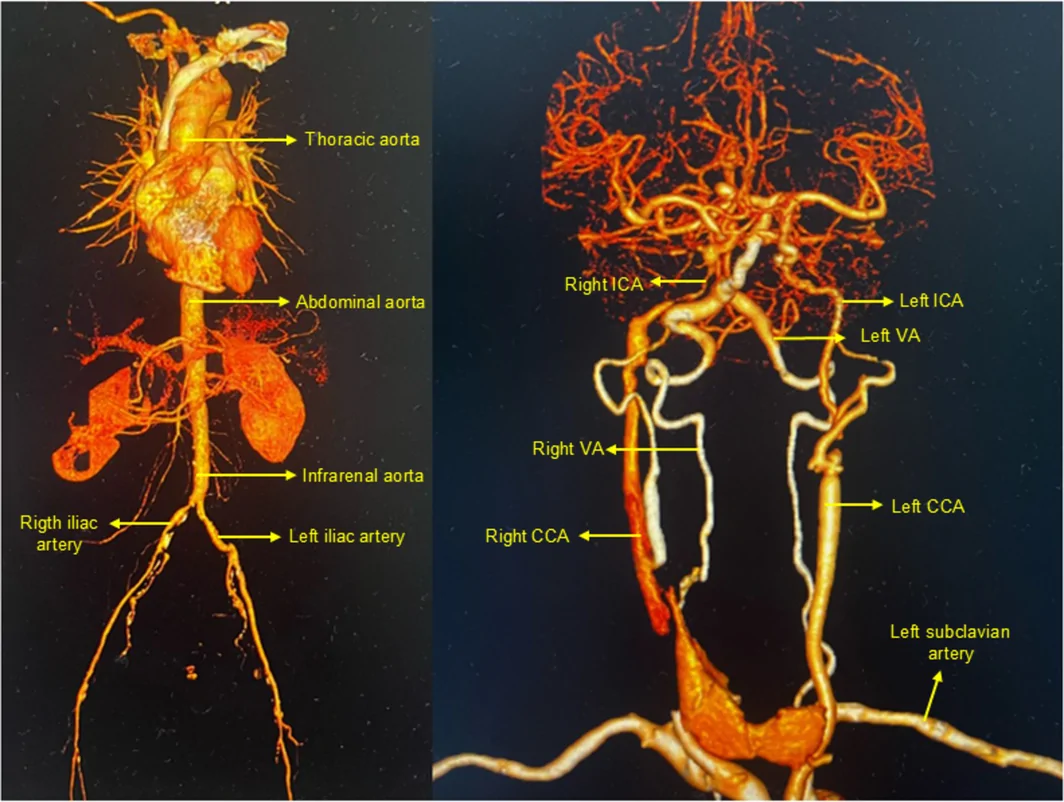
Three‐dimensional computed tomography angiography demonstrates diffuse, long‐segment vascular involvement affecting the aorta and multiple major branches, with heterogeneous luminal remodeling and asymmetric arterial changes, consistent with a large‐vessel vasculopathy pattern. CCA, common carotid artery; ICA, internal carotid artery; VA, vertebral artery.

After 7 months, during follow‐up, a new vascular neuroimaging study evidenced occlusion of the P2 PCA segment. At this moment, a thoracic and abdominal CTA was performed. As demonstrated in the three‐dimensional reconstructions (Figure 2), diffuse and multiterritorial involvement of large vessels is observed. Although the aorta has a preserved caliber, its thoracic and infrarenal portions exhibit irregularities in the luminal contour, suggestive of parietal thickening. Both iliac arteries, as well as the left subclavian artery, present hemodynamically significant stenoses. The renal arteries show diffuse luminal reduction, without relevant stenoses. A mixed plaque was identified at the origin of the celiac trunk, responsible for severe stenosis (> 70%) with preservation of the patency of the mesenteric arteries. No other atheromatous plaques or calcifications were described. Arterial Doppler ultrasound of the lower limbs showed occlusion or near‐occlusion of the right posterior tibial artery and the left superficial femoral artery.

Taken together, these findings indicated a systemic, diffuse, and multiterritorial steno‐occlusive arterial disease, with a morphological and distributional pattern favoring large‐vessel vasculitis, particularly Takayasu arteritis, characterized by involvement of the aorta and its major branches, including supra‐aortic, abdominal, and peripheral arteries, long and non‐eccentric stenoses, diffuse luminal irregularities suggestive of inflammatory wall thickening, and absence of typical features of advanced atherosclerosis.

Extracranial duplex ultrasound demonstrated occlusion of the ICA from its origin, as well as a > 50% stenosis at the origin of the external carotid artery (ECA). The right vertebral artery (VA) was patent and showed proximal hypoechoic wall thickening measuring 2.1 mm, associated with marked flow turbulence and increased peak systolic velocity (PSV: 115 cm/s), consistent with a hemodynamically significant stenosis (> 50%). The right subclavian artery exhibited hyperechoic wall thickening at its origin measuring 4.2 mm, with a PSV of 117 cm/s, compatible with < 50% stenosis.

On the left side, the CCA showed diffuse, circumferential hyperechoic wall thickening measuring 1.7 mm. At the proximal segment, the ICA presented hypoechoic wall thickening measuring 2.2 mm, with marked turbulence and Doppler features of near‐occlusion, including monophasic low‐velocity flow in the mid segment (PSV: 22 cm/s). The left VA was patent, with wall thickening and a hemodynamically significant stenosis (> 50%). The left subclavian artery showed wall thickening measuring 1.0 mm, with only a mild increase in PSV.

**FIGURE 3:**
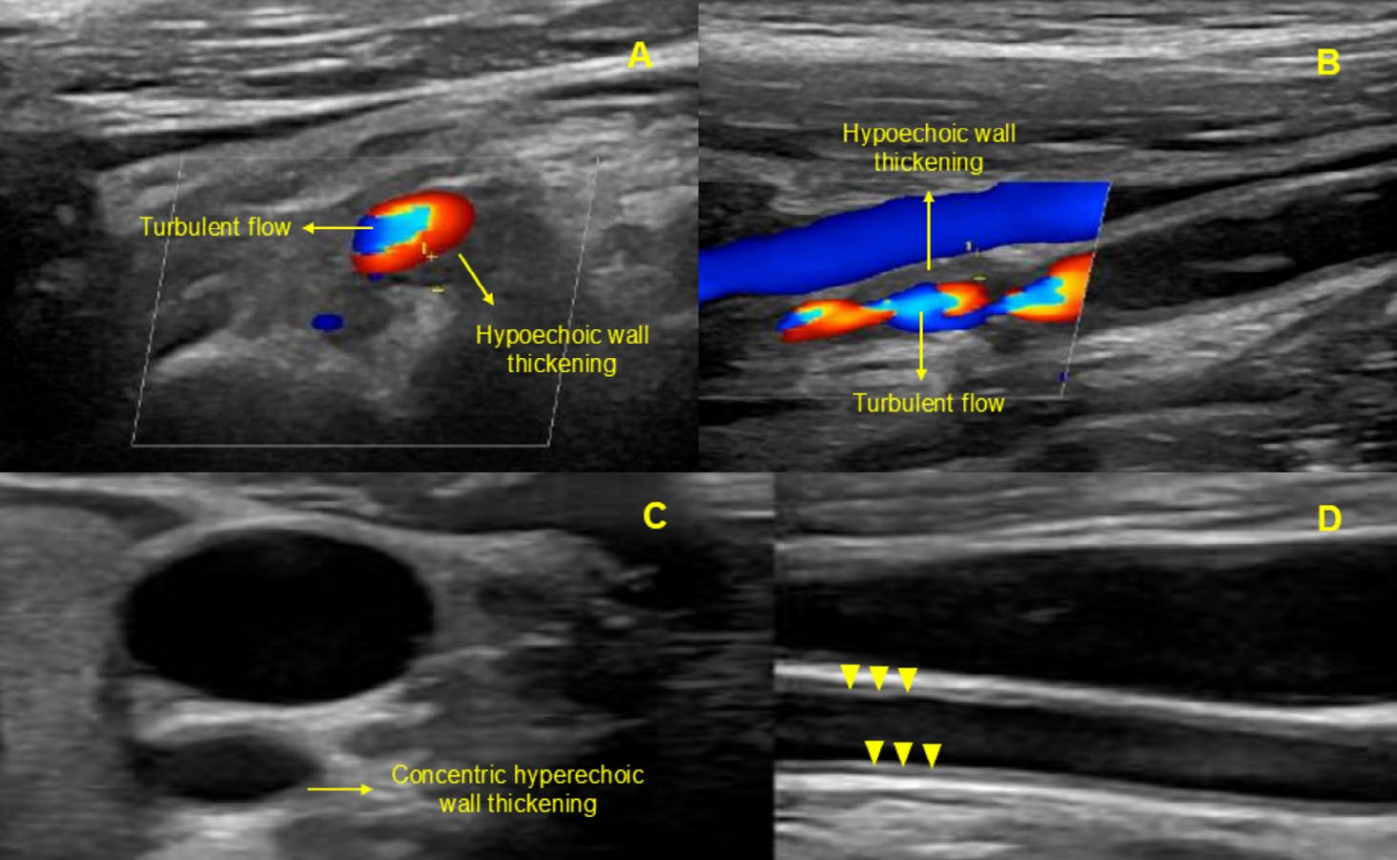
Doppler ultrasound of the cervical vessels in a patient with large‐vessel vasculitis. (A, B) B‐mode and color Doppler images show hypoechoic circumferential arterial wall thickening (arrows) with associated luminal turbulent flow, findings compatible with inflammatory arterial wall involvement. (C, D) Homogeneous concentric hyperechoic wall thickening (arrowheads), compatible with chronic post‐inflammatory arterial wall remodeling.

Overall, this pattern of multiterritorial wall thickening and stenoses suggested ongoing inflammatory vascular involvement, predominantly affecting the left ICA and right VA, as illustrated by the hypoechoic wall thickening shown in Figure 3. Figure 3 also demonstrates concentric wall thickening of the CCA, compatible with chronic post‐inflammatory arterial remodeling.

Temporal artery ultrasound was not performed, as the patient did not present cranial symptoms suggestive of cranial giant cell arteritis, including headache, scalp tenderness, or jaw claudication.

The patient subsequently developed bilateral visual impairment due to left central retinal artery occlusion and right anterior ischemic optic neuropathy (AION). She was referred for multidisciplinary evaluation, including rheumatology and neurology, and giant cell arteritis was considered given the ophthalmological and intracranial involvement. However, the overall clinical presentation and vascular imaging pattern were not consistent with isolated cranial involvement, and the final diagnosis of Takayasu arteritis was established. Pulse therapy with methylprednisolone was then initiated.

**FIGURE 4:**
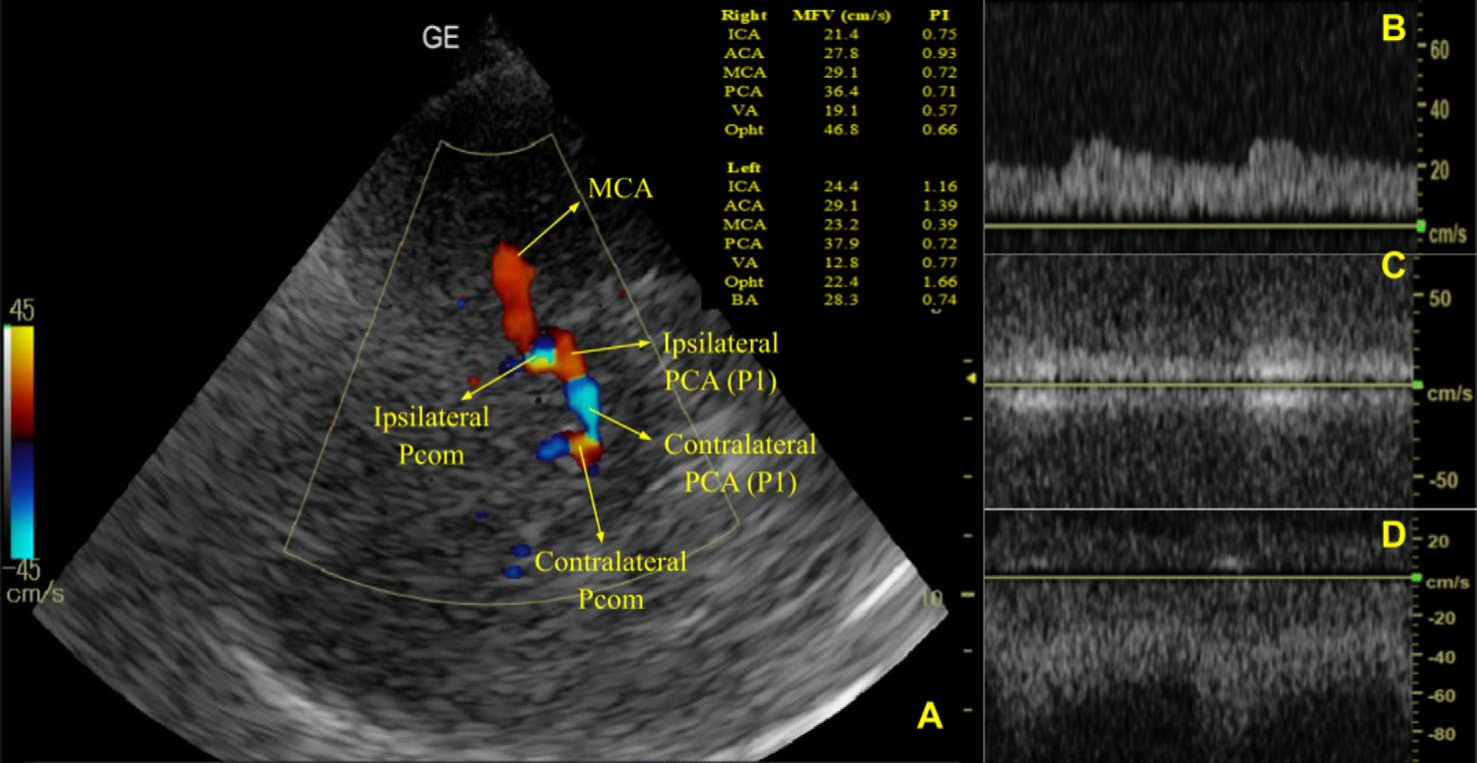
Transcranial color‐coded duplex (TCCD) sonography in Takayasu arteritis. A: TCCD transtemporal window showing Pcom activation with aliasing artifact; B: Flattening of the MCA spectral curve, with reduction of the pulsatility index (PI) at left; C: Turbulent flow in the right petrous segment of ICA; D: Pcom spectral curve with increased flow. ACA, anterior cerebral artery; BA, basilar artery; ICA, internal carotid artery; MCA, middle cerebral artery; MFV, mean flow velocity; Opht, ophthalmic artery; Pcom, posterior communicating artery; VA, vertebral artery.

To better understand the intracranial perfusion pattern, TCCD imaging was performed (Figure 4). It showed reduced velocity and a flattened spectral curve in the middle cerebral arteries (MCAs). Also, the pulsatility index (PI) of the left MCA is low (< 0.6). Turbulent flow was observed in the right petrous segment of the ICA, compatible with hemodynamically significant stenosis. In color‐coded duplex imaging, activation of the right posterior communicating artery (Pcom) is visible, with aliasing artifacts due to functional stenosis. Although there is an increase in diameter of the vertebrobasilar system, velocities were not changed. Both ophthalmic arteries had anterograde flow, but there was a reduction in PI at the right. A breath‐holding test was not performed because the patient developed cognitive impairment after the ischemic event, precluding adequate cooperation with the maneuver.

The TCCD findings were corroborated by CTA, as described previously. Digital subtraction angiography was not performed, as CTA provided sufficient anatomical detail for diagnostic and clinical purposes.

## 3 Diagnostic Considerations and Classification

Histopathological confirmation was not obtained, as biopsy is rarely feasible or diagnostic in large‐vessel vasculitis involving deep arterial segments. The final diagnosis was established after multidisciplinary evaluation, based on clinical presentation and extensive imaging findings.

According to the 2022 ACR/EULAR classification criteria for TA, the patient fulfills several key domains, including female sex, extensive involvement of the aorta and its major branches (subclavian, carotid, vertebral, renal, iliac, and lower‐limb arteries), and a disease distribution pattern compatible with large‐vessel vasculitis (Dejaco et al. 2018).

The patient was treated with high‐dose intravenous corticosteroids, followed by oral prednisone. Methotrexate was subsequently introduced as a corticosteroid‐sparing agent, allowing for gradual tapering of prednisone. Since initiation of immunosuppressive therapy, no new ischemic cerebrovascular events have occurred, and the patient's neurological status has remained stable throughout follow‐up.

Table 1 summarizes the main clinical, laboratory, and imaging features supporting the diagnostic adjudication in this case.

**TABLE 1:** Comparative clinical and imaging features of large‐vessel vasculopathies and findings in the present case.

Feature	Takayasu arteritis	Giant cell arteritis	Atherosclerosis	Findings in the present case
Typical age at onset	≤ 50 years (overlap with early GCA)	> 50 years	Variable, increases with age	50 years
Sex predominance	Female	Female	Male predominance	Female
Constitutional symptoms	Common, but may be absent	Common	Absent	Absent
ESR/inflammatory markers	May be normal	Usually elevated (may be normal in a minority)	Normal	Normal ESR
Vessel distribution	Aorta and major branches	Extracranial carotid branches, aorta	Focal large and medium arteries	Diffuse aortic and multibranch involvement
Pattern of arterial disease	Diffuse wall thickening, long segments	Segmental inflammation	Focal plaques	Long, multiterritorial involvement
Intracranial involvement	Increasingly recognized	Uncommon	Possible	Present
Vertebrobasilar dolichoectasia	Very rare/scarcely reported	Not typical	Possible but uncommon	Present
Collateral circulation	Prominent	Variable	Variable	Marked (Circle of Willis)
Response to steroids	Expected	Expected	No	Clinical and imaging stabilization after treatment

The overall vascular involvement encompassed multiple large‐artery territories, including the carotid, vertebral, subclavian, aorta, iliac arteries, and lower‐limb vessels. This diffuse and multiterritorial distribution is more consistent with a large‐vessel vasculitis pattern than with isolated or accelerated atherosclerosis.

## 4 Discussion

Stroke is uncommon in patients with TA, given the slow progression of extracranial stenosis, which allows the establishment of collaterals and the maintenance of cerebral flow and metabolism until more advanced stages (Fauci 1994; Takano et al. 1993). Therefore, previous studies have recommended the use of functional and hemodynamic assessments of these patients, rather than anatomical images, to identify those at risk of ischemia (Hoffmann et al. 2000; Lee et al. 2003).

The so‐called “non‐pulsatile cerebral perfusion” pattern reflects a hemodynamic consequence of extensive proximal large‐vessel disease, rather than a disease‐specific marker. Similar flow patterns may be observed in other conditions, including severe bilateral atherosclerotic carotid disease, bilateral dissections, and other forms of large‐ or medium‐vessel vasculitis (Lee et al. 2003; Ameriso et al. 1990). The “non‐pulsatile cerebral perfusion”, with reduced mean flow velocities, flattened curve spectra (blunted), and reduced PI, mainly in the arteries of the anterior circulation, may be observed in patients with TA (Ameriso et al. 1990). These findings correspond to the post‐stenotic pattern secondary to carotid involvement. There is also a predominance of primary collateralization by the circle of Willis (Hoffmann et al. 2000).

Atherosclerosis represents an important differential diagnosis, particularly in a patient with cardiovascular risk factors such as hypertension and smoking history. However, in the present case, the pattern of arterial involvement was diffuse and multisegmental, extending beyond typical atherosclerotic predilection sites and involving supra‐aortic, abdominal, pelvic, and lower‐limb arteries. Moreover, imaging findings were not dominated by focal calcified plaques at arterial bifurcations, but rather by long‐segment stenoses and occlusions affecting multiple vascular territories, supporting a vasculitic rather than a purely atherosclerotic process (Sawalha et al. 2025).

Anterior ischemic optic neuropathy, while common in hypertensive patients, was interpreted as a supportive but non‐specific finding within the broader context of systemic vascular disease (Hayreh 2009).

Although CT and MRI remain the reference imaging modalities for large‐vessel vasculitis, ultrasonography has demonstrated the ability to detect clinically relevant structural and hemodynamic abnormalities in major vascular districts, including the aorta and its main branches. Ultrasound allows real‐time assessment of vessel wall alterations and flow patterns, facilitating early diagnostic suspicion, even during examinations performed for other clinical indications, and guiding appropriate referral to second‐level imaging techniques. In large‐vessel vasculitis, Doppler‐derived functional information may meaningfully complement the predominantly morphological data obtained from CT or MRI (Germanò et al. 2017; Corvino et al. 2024).

Ultrasound assessment of the extracranial carotid and vertebral arteries has also been shown to play a key role in the evaluation of acute cerebral ischemia, enabling the detection of stenosis, occlusions, and abnormal flow patterns that may reflect underlying large‐vessel pathology and guide further imaging decisions (Germanò et al. 2017; Psychogios et al. 2020). In the presence of inflammatory activity from vasculitis, ultrasound may show hypoechoic circumferential thickening of the vessel wall, consistent with parietal edema, characterizing the halo sign. With response to treatment and regression of the inflammatory process, the arterial wall tends to become progressively more hyperechoic and homogeneous, configuring the so‐called macaroni sign (Keo et al. 2013).

The presence of intracranial stenosis with circumscribed areas of turbulence, as in our patient's case, is not frequently mentioned in the literature (Hoffmann et al. 2000). The focus of extracranial involvement as a cause of neurological injury may have overshadowed research and investigation of the intracranial pattern of involvement (Yahya et al. 2023). However, advances in neuroimaging studies have made intracranial involvement in TA more clearly established. Cerebral vasculitis may occur in up to 7.8% of cases, and stroke secondary to occlusion of large intracranial vessels in 3.9% (Bond et al. 2017).

Ectasia in vessels other than the aorta is rare and is a previously described angiographic finding in coronary arteries (Endo et al. 2003). To our knowledge, there are no previous reports describing dolichoectasia of intracranial vessels, especially of the vertebrobasilar system, and the present case is a rare presentation.

## 5 Limitations

This report has several limitations inherent to its observational and retrospective nature. First, histopathological confirmation was not available, and the diagnosis of TA was established based on the overall clinical, laboratory, and multimodal imaging profile, supported by current ACR/EULAR classification criteria. Although this approach reflects real‐world clinical practice, particularly in large‐vessel vasculitis, it limits definitive etiological confirmation. In addition, inflammatory markers were normal at presentation, which may have reduced early clinical suspicion and delayed a unifying diagnosis.

From an imaging standpoint, although extracranial duplex ultrasound provided detailed morphological and hemodynamic information across multiple vascular territories, a dedicated vessel wall ultrasound protocol specifically designed to systematically characterize classic signs such as the halo or macaroni sign was not prospectively applied. The ultrasound examinations were performed as part of routine clinical assessment rather than a standardized research protocol. Consequently, while concentric circumferential wall thickening with different echogenic patterns, suggestive of active inflammation and chronic post‐inflammatory remodeling, could be retrospectively identified and illustrated, a textbook, long‐segment, smooth, and homogeneous vessel wall appearance cannot be uniformly guaranteed across all examined arteries.

Finally, some functional assessments of cerebrovascular reserve, such as breath‐holding or cuff‐induced hyperemia tests, were not performed, limiting a more comprehensive evaluation of cerebrovascular reactivity. Despite these limitations, the convergence of clinical evolution, diffuse multiterritorial vascular involvement, absence of typical advanced atherosclerotic features, and consistent multimodal imaging findings supports the diagnosis of large‐vessel vasculitis and highlights the complementary role of ultrasound within an integrated diagnostic approach.

## 6 Conclusion

This case highlights the value of TCDD in revealing complex intracranial hemodynamic adaptations in patients with extensive large‐vessel disease. While not diagnostic in isolation, these findings may raise suspicion for underlying vasculitis and support timely referral for comprehensive vascular evaluation.

## Funding

The authors have nothing to report.

## Ethics Statement

This case report was conducted in accordance with the ethical standards of the institutional and national research committees and with the 1964 Helsinki Declaration and its later amendments. Ethical approval was not required for this single‐patient case report, per our institution's policies.

## Consent

Written informed consent was obtained from the patient for publication of this case report and any accompanying images. All identifying information has been omitted to ensure patient confidentiality.

## Conflicts of Interest

The authors declare no conflicts of interest.

## Data Availability

All relevant data supporting the findings of this study are included within the article. Additional information is available from the corresponding author upon reasonable request.
